# Genetics of Polyketide Metabolism in *Aspergillus nidulans*

**DOI:** 10.3390/metabo2010100

**Published:** 2012-01-30

**Authors:** Marie L. Klejnstrup, Rasmus J. N. Frandsen, Dorte K. Holm, Morten T. Nielsen, Uffe H. Mortensen, Thomas O. Larsen, Jakob B. Nielsen

**Affiliations:** 1 Department of Systems Biology, Center for Microbial Biotechnology, Technical University of Denmark, Søltofts Plads B221, DK-2800 Kgs. Lyngby, Denmark; Email: marlk@bio.dtu.dk (M.L.K.); tol@bio.dtu.dk (T.O.L); 2 Department of Systems Biology, Center for Microbial Biotechnology, Technical University of Denmark, Søltofts Plads B223, DK-2800 Kgs. Lyngby, Denmark; Email: rasf@bio.dtu.dk (R.J.N.F.); dmkp@bio.dtu.dk (D.K.H.); motni@bio.dtu.dk (M.T.N.); um@bio.dtu.dk (U.H.M.)

**Keywords:** secondary metabolites, polyketides, polyketide synthases, gene clusters, biosynthesis, *Aspergillus nidulans*

## Abstract

Secondary metabolites are small molecules that show large structural diversity and a broad range of bioactivities. Some metabolites are attractive as drugs or pigments while others act as harmful mycotoxins. Filamentous fungi have the capacity to produce a wide array of secondary metabolites including polyketides. The majority of genes required for production of these metabolites are mostly organized in gene clusters, which often are silent or barely expressed under laboratory conditions, making discovery and analysis difficult. Fortunately, the genome sequences of several filamentous fungi are publicly available, greatly facilitating the establishment of links between genes and metabolites. This review covers the attempts being made to trigger the activation of polyketide metabolism in the fungal model organism *Aspergillus nidulans*. Moreover, it will provide an overview of the pathways where ten polyketide synthase genes have been coupled to polyketide products. Therefore, the proposed biosynthesis of the following metabolites will be presented; naphthopyrone, sterigmatocystin, aspyridones, emericellamides, asperthecin, asperfuranone, monodictyphenone/emodin, orsellinic acid, and the austinols.

## 1. Introduction

*Aspergillus nidulans*, teleomorph *Emericella nidulans*, is one of the most significant biological model systems in the fungal kingdom. This was pioneered by Pontecorvos’ [1] work in the middle of the last century, which demonstrated that *A. nidulans*, in addition to the asexual state, also proliferate via sexual and parasexual life cycles, hence, offering an ideal platform for genetic studies. Related species in the genus *Aspergillus* include important industrial cell factories, *A. niger* and *A. oryzae*, species that cause allergic diseases, *A. clavatus*, as well as opportunistic pathogens, such as *A. fumigatus*.

A common feature of aspergilli and filamentous fungi in general is their capacity to produce secondary metabolites (SMs). As opposed to the primary metabolites, SMs are not essential for cellular growth, but provide fungi, as well as bacteria and plants, with a competitive advantage in nature, e.g., by serving as agents for chemical warfare or as signal molecules. Hence, an impressive range of compounds with broad ranging bioactivities has evolved. SMs can be divided into four main chemical classes: Polyketides (PK), terpenoids, shikimic acid derived compounds, and non-ribosomal peptides (NRP). Moreover, hybrid metabolites composed of moieties from different classes are common, as in the meroterpenoids, which are fusions between PKs and terpenes. Hybrid molecules significantly add to the complexity and variety of the fungal metabolomes.

In addition to their likely important ecological roles in their natural biological niches, SMs also have a considerable impact on human life. For instance aflatoxins, ochratoxins, and fumonisins act as mycotoxins by having a detrimental effect on humans and livestock, whereas others are beneficial and serve as food additives, pigments, cholesterol-lowering drugs, immunosuppressants, antibiotics and anticancer agents. The different aspects of SM action and application have spurred a tremendous interest in fungal secondary metabolites, which is further underlined by the fact that around 63% of all small molecule drugs, which reached the market from 1981–2006 were inspired by natural products or derivatives thereof [2].

In filamentous fungi, the competitive race in SM development and the cost of producing and secreting complex compounds have resulted in the evolution of a multifaceted regulation of SM biosynthesis to avoid unnecessary use of resources. This hampers their discovery since production of most SMs is not induced under laboratory conditions. Analysis of full genome sequences of eight different aspergilli have demonstrated that for the majority of genes that putatively encode enzymes for SM production, the product is not known or detected. In this review, we will provide highlights of the use of genome mining, sophisticated molecular biological and chemical tools to trigger the production of SMs from cryptic gene clusters and discuss how these techniques have accelerated our understanding of PK production and regulation in *A. nidulans*.

### 1.1. Polyketide Biosynthesis in A. nidulans

PKs in fungi are synthesized by the use of acyl-CoA units. They act as the general substrates for large multi-domain enzymes named polyketide synthases (PKSs), which resemble eukaryotic fatty-acid synthases (FASs) in domain architecture. PKSs are divided into three types of PKSs based on their catalytic organization, however, only the iterative type I PKS (iPKS) has been reported in *A. nidulans*. The iPKS repeat the use of a single module containing several catalytic domains until the growing chain of acyl-CoA units block further elongation. For descriptions of PKSs in general, excellent reviews by Crawford [3], Hertweck [4] and Cox [5] can be consulted. The most commonly encountered catalytic activities in fungal PKSs will be addressed as a general introduction to fungal PKSs in the following three paragraphs.

Three fundamental domains are found in all iPKSs in *A. nidulans* like in filamentous fungi in general; β-ketosynthase (KS), acyltransferase (AT), and the acyl carrier protein (ACP). The KS catalyzes the C–C bond formation via decarboxylation reactions through Claisen condensations between thioesters. The ACP domain is responsible for transiently holding the growing acyl chain, hereby allowing the loading of malonyl extender units. The acyl groups are transferred from CoA by AT onto KS and ACP. The iterative use of the three domains results in a non-reduced PK, a β-keto thioester. Additional domains can be present in the PKS allowing the introduction of further chemical complexity.

iPKSs in fungi can, based on their catalytic domains, be classified as non-reducing (NR-PKSs), partially reducing (PR-PKSs), or highly reducing (HR-PKSs) [6]. This is based on their ability to reduce the β-keto carbon. In PR- and HR-PKSs, reduction occurs through the β-ketoreductase (KR) domain that converts the β-ketone to a hydroxyl group. The resulting hydroxyl can go all the way to saturation by elimination of water through the dehydratase (DH) domain followed by hydrogenation by enoyl reductase (ER). In addition, reducing PKSs can also possess a methyltransferase domain (MT) responsible for C-methylation of the growing PK chain, using S-adenosylmethionine (SAM) as a carbon-donor. The degree of modifications and their position in the PK product is always the same for the individual PKSs. However, it is presently unknown how deployment of the various modifying domains is programmed into the PKS enzyme.

NR-PKSs differ in domain architecture from reducing PKS by not having any of the reducing domains and by having an N-terminal starter unit-ACP transacylase (SAT) domain and an internal product template (PT) domain. The SAT domain is responsible for selecting the starter unit to be extended by the enzyme [7], while the PT domain is responsible for folding and cyclization of the non-reduced PK backbone [3,8]. The number of iterations within the PKS and thereby the display of functional groups and the size of the final product is likely determined by the size of the active site cavity in the iPKS [9]. Once the length of the final product has been achieved, the PK chain is released from the PKS, catalyzed by either a thioesterase (TE), a Claisen cyclase (CLC) domain if present, or by accessory enzymes. A more detailed discussion on PKS release mechanisms is reviewed by Du and Lou [10].

It should be noted that it currently is impossible to reliably predict the product of iPKSs based on their amino acid sequences and domain architecture. This is in part due to the inability to predict the number of iterations performed by the iPKS and in part due the lack of understanding of how deployment of tailoring domains in individual iterations are programmed into the enzyme.

Interestingly, the PKS encoding gene tends to reside in clusters of genes coding for a broad range of enzymatic activities. The compound coming directly from the PKS rarely seem to be the final product in the biosynthesis, but usually undergoes further modifications by tailoring enzymes from small decorations to drastic and large intervention and couplings.

**Figure 1:**
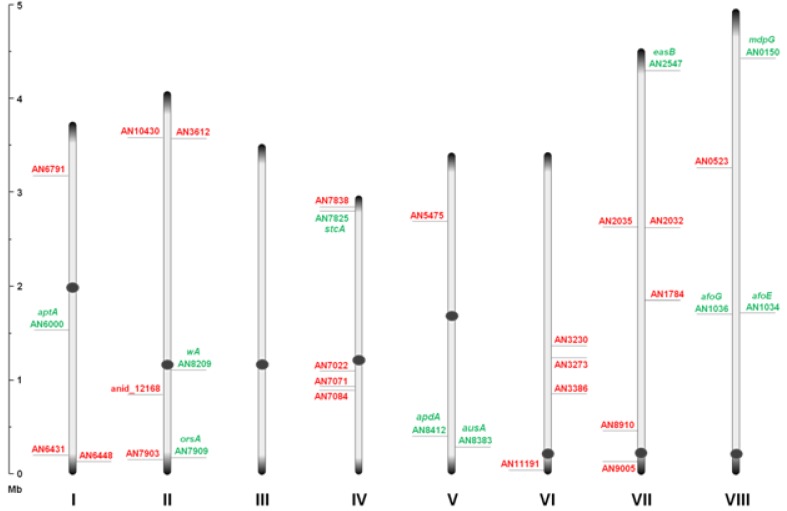
An overview of the relative distribution of the 32 putative polyketide synthases (PKS) open reading frames (ORFs) on the eight chromosomes of *A. nidulans*. Green and red AN numbers represent assigned and unassigned PKS genes, respectively. Dark grey circles and ends symbolize centromeric and telomeric regions, respectively, and should not be considered to scale.

Through inspection of the genome sequence (genome mining), the latest estimate of genes encoding PKSs in *A. nidulans* is 32 open reading frames (ORFs) [11] (Figure 1), indicating that the number of PK containing end products in *A. nidulans* should count at least 32 plus stable intermediates. The compounds detected under a given condition do not necessarily reflect the final outcome of a PK pathway, since the presence of intermediates and shunt products depends on other downstream enzymes and regulation.

At present, a total of nine PKS genes have been coupled to the polyketome (collection of PKs and their synthesis) in *A. nidulans*, and numerous endeavors are currently attempting to unveil the mechanisms of PK metabolism in this model fungus. The pathways described in this review will follow in chronological order with respect to the discovery from PK to genes. For each of the PK gene clusters that have been linked to products so far we will focus on the PK compounds, their discovery, genetics as well as their biosynthetic pathway: Naphthopyrone, sterigmatocystin, aspyridones, emericellamides, asperthecin, asperfuranone, monodictyphenone (emodin), orsellinic acid, and the austinols.

## 2. Naphthopyrone

Spores from *A. nidulans* are characterized by a dark grey-green macroscopic appearance. This is due to deposition of pigments in the conidial wall as shown by ultrastructure studies using transmission electron microscopy (TEM) [12]. The responsible pigment is based on the PK-naphthopyrone YWA1 and the function of the pigment layer has been shown to include quenching of reactive oxygen species [13] and increased resistance to UV radiation [14]. The work on naphthopyrone synthesis in *A. nidulans* has paved the way for understanding iPKS domain structure.

The study of conidial pigmentation in *A. nidulans* has been extremely valuable for genetic screens. The first pigment mutant recorded in literature was the spontaneous white alba (*w_a_*) strain reported by Yuill in 1939 [15]. In addition to the white mutant class, *yA^−^* mutants producing yellow conidia were discovered. These available color variants served as easy recognizable markers (green, white, and yellow) that allowed the establishment of fundamental genetic tools in *A. nidulans* [1]. Interestingly, sexual crossing showed that the *wA*^−^ mutation masked the effect of the *yA*^−^ mutation (epistatic) [1,16]. Clutterbuck and co-workers [16] proposed that WA synthesized the yellow pigment that was observed in *yA* mutants and that the YA enzyme converted this compound into the green conidial pigment. In 1967, Agnihotri and co-workers [17] found that the wild type strains if grown under copper limiting conditions could mimic the yellow phenotype of the *yA^−^* strain. *yA* (AN6635) and *wA* (AN8209) were isolated and mapped to loci, chromosome I and II respectively, by complementation of a cosmid based library in 1989 and 1990, respectively [18,19]. Later, cross-feeding experiments performed by Clutterbuck [16] revealed that the *yA* phenotype was caused by the lack of a copper dependent extracellular laccase (p-diphenol oxidase). The *wA* functionality in pigment formation was confirmed by gene-deletion studies [19]. The lack of clustering of the two *A. nidulans* conidial pigment genes also became evident by their different expression patterns and the finding that they are controlled by different regulatory systems [20,21]. The *yA* gene is expressed in phialide cells and primary sterigmata (metulae) [18], and controlled by BrlA and AbaA [21], while *wA* is expressed only in phialides [22] and controlled by WetA [20]. Interestingly, none of the genes are expressed in the conidia. Characterization of the WA PKS was accomplished by Northern blotting, which revealed that *wA* encoded a 7.5 kb large transcript [19], and sequencing of the locus [22]. Re-sequencing of the 3′ region in 1998 led to a revised gene model of the PKS with the following domain structure KS-AT-ACP-CLC. This novel CLC domain [23] catalyzed release of the product and cyclization of the second aromatic ring of YWA1 via a Claisen condensation reaction [24].

**Figure 2:**
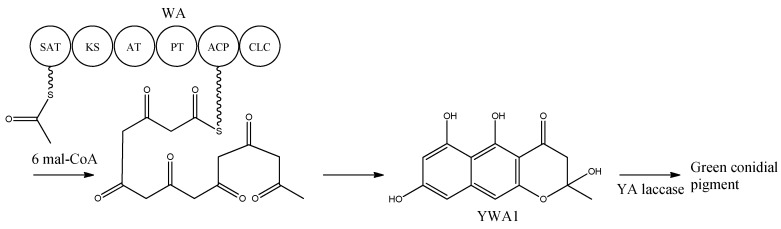
Biosynthetic pathway for formation green conidial pigment in *A. nidulans*

Heterologous expression of *wA* in *A. oryzae* resulted in the production of the yellow compound, as observed in *yA^−^* mutants, which was identified to be the heptaketide naphthopyrone named YWA1 [25]. In 2002, *wA* was used for constructing a collection of chimeric PKSs (cPKS) by mixing its domains with those of *Colletotrichum lagenarium pks1*, known to produce the tetraketide 1,3,6,8-tetrahydroxynaphthalene (T4HN). One of the resulting cPKSs, SW-B, produced several new compounds including both *tetra*- and pentaketides [26]. The results prompted a reanalysis of the two PKSs, which revealed the existence of two previously overlooked conserved domains; an N-terminal and a central domain. These domains were later identified as a SAT and PT domain, respectively, thus providing the full domain structure SAT-KS-AT-PT-ACP-CLC [3,7,8]. With the organization within WA in mind, the biosynthetic pathway can be envisioned as condensations of an acetyl-CoA with six malonyl-CoA units in six successive reactions resulting in the formation of YWA1 [25] (Figure 2).

YWA1 is then believed to be dimerized or polymerized by the YA laccase into the green conidial pigment(s) via phenolic oxidative coupling. However, to date no one has succeeded in characterizing the chemical structure of the green conidial pigmentation in detail. As the final product remains elusive, it is impossible to predict if other tailoring enzymes further modify the YWA1 backbone or reactions occur with other metabolites or cellular components, e.g., the cell wall.

## 3. Sterigmatocystin

Sterigmatocystin, a PK, was first partially purified from a *Sterigmatocytis* sp. in 1948 by Nekam and Polgar [27]. Hatsuda and co-workers [28,29] successfully isolated sterigmatocystin in 1954 from *A. versicolor*. The correct relative structure was determined in 1962 by Bullock *et al.* [30]. By performing degradative experiments it was shown that the stereochemistry of sterigmatocystin was the same as that of aflatoxin [31], which had the absolute stereochemistry determined in 1967 [32]. The absolute stereochemistry of sterigmatocystin was confirmed via crystallography [33,34].

The aflatoxins are among the most carcinogenic mycotoxins and the research in aflatoxin and sterigmatocystin intensified with the Turkey X disease caused by aflatoxins in the middle of the last century [35]. Aflatoxins are reported to be produced only by a few aspergilli. *A. nidulans* does not produce aflatoxins, as the biosynthesis stops at sterigmatocystin, a late, yet stable precursor of the pathway. Sterigmatocystin is a powerful mycotoxin, though it is estimated to be 150 times less carcinogenic than the most potent aflatoxin, B_1_ [36]. Fungi that are able to produce aflatoxins and/or sterigmatocystin are common contaminants of food, feed, and indoor environments and may be mammalian and plant pathogens [37,38]. Due to the high toxicity and prevalence of sterigmatocystin and aflatoxins, they are likely the most extensively studied examples of secondary metabolism in fungi both in terms of biosynthesis and biological function, and there are several excellent and comprehensive reviews for further reading on aflatoxin biosynthesis [39,40]. Studies on the biosynthesis of aflatoxin and sterigmatocystin have been carried out in several fungi (*A. flavus*, *A. nidulans* and *A. parasiticus*) and some of the assigned gene functions in *A. nidulans* are proposed based on gene homology to these two other species.

**Figure 3:**
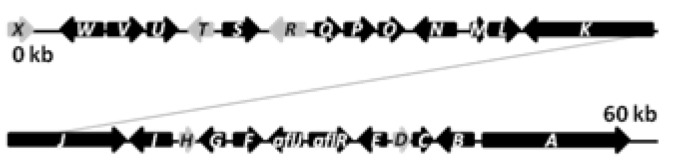
The sterigmatocystin gene cluster. The black arrows are predicted *stc* ORFs, while light grey arrows are genes with unassigned functions.

The biosynthetic cluster of sterigmatocystin in *A. nidulans* was first characterized by Brown and co-workers in 1996 [41]. They identified a 60 kb region in the *A. nidulans* genome responsible for the synthesis of sterigmatocystin. The cluster contains 27 genes named s*tcA-X* (Figure 3), reflecting their order of appearance on the chromosome [41].

The PKS catalyzing the production of the PK backbone of sterigmatocystin was identified by Yu and co-workers in 1995 [42] and originally named *pksST*, but was later renamed to *stcA* by Brown *et al.* [41] to simplify nomenclature. Besides the PKS, the *stc* gene cluster is predicted to contain two transcription factors (*aflR*, *aflJ*), six monoxygenases (*stcB*, *stcC*, *stcF*, *stcL*, *stcM*, *stcS*, *stcW*), two dehydrogenases (*stcG*, *stcV*), an esterase (*stcI*), an O-methyltransferase (*stcP*), two ketoreductases (*stcE*, *stcU*), a VERB synthase (*stcN*), an oxidase (*stcO*), a monooxydase/oxidase (*stcQ*), a Baeyer-Villiger oxidase (*stcR*), a fatty acid synthase composed by the two subunits HexA and HexB (encoded by *stcJ* and *stcK*, respectively), and five unassigned genes (*stcD*, *stcH*, *stcR*, *stcT*, *stcX*), which may also be part of the cluster [39,40,41,43,44,45,46,47,41,43].

The *stc* cluster is a relatively large gene cluster, and studying the gene regulation has led to important discoveries. The two TFs were found to be present within the cluster. The AflR is a Zn_2_Cys_6_ TF that regulates transcription of the *stc* locus in *A. nidulans* [41,48], while AflJ (also named AflS) have been shown to have a role in the regulation of aflatoxin biosynthesis in *A. flavus* and is likely to have a similar function in *A. nidulans* [49]. Interestingly, Bok and Keller [50] discovered a novel regulator (LaeA) of secondary metabolism in *A. nidulans* in a mutant screen for loss of *aflR* expression. Deletion of *laeA* resulted in a significantly decreased production of different classes of SMs like sterigmatocystin and penicillin. LaeA, a putative methyl transferase was moreover acting in a feedback loop with AflR since overexpression of *aflR* downregulates *laeA* expression, and overexpression of *laeA* could not increase production of sterigmatocystin [50]. LaeA was shown to be a part of the conserved Velvet complex, which is important for regulation of fungal development and secondary metabolism [51]. Another hint on chromatin regulated gene expression came from the deletion of a histone deacetylase, *hdaA*Δ, which led to significant increase in the expression of two *stc* cluster genes, *stcU* and *aflR* compared to the reference [52].

Applying this strategy of deleting and overexpressing genes encoding global epigenetic regulators has paved the way for novel discoveries in secondary metabolism. Moreover the alternative of utilizing a chemical epigenetic approach through epigenetic modifier molecules has proven successful in activating gene clusters in *A. niger* [53].

**Figure 4:**
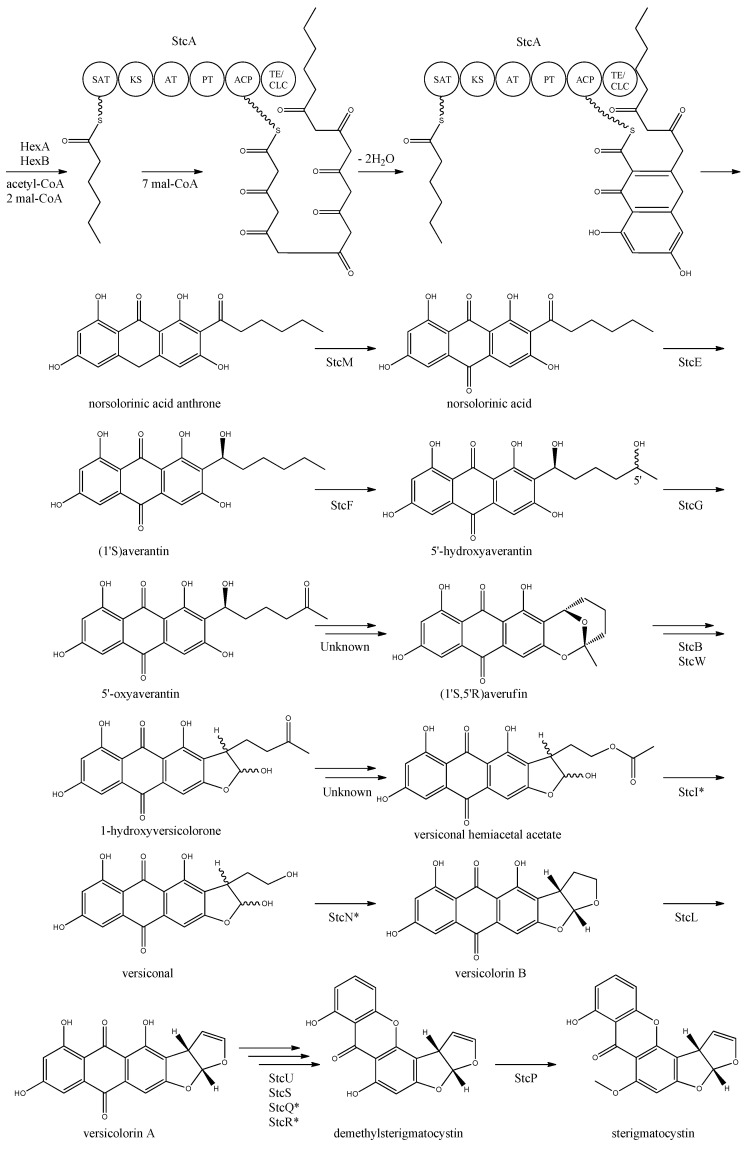
Proposed biosynthesis of sterigmatocystin. StcA contains starter unit-ACP transacylase (SAT), β-ketosynthase (KS), acyltransferase (AT), product template (PT), acyl carrier protein (ACP) and thioesterase/claisen cyclase (TE/CLC) domains. *Indicates a proposed, but not confirmed, enzyme. Multiple arrows indicate that the number of enzymatic steps is unknown.

The first step in the biosynthesis of sterigmatocystin (Figure 4) is the production of hexanoate by the FAS units, StcJ and StcK [41]. Watanabe and Townsend [54] showed that the hexanoyl-CoA is not an intermediate freed from the complex, indicating that hexanoate is transferred directly to the SAT domain of the PKS. The PK backbone is assembled by StcA by condensation of the starter unit, hexanoyl-CoA and seven malonyl-CoA extender units followed by cyclization and release of norsolorinic acid anthrone [42]. The oxidation of norsolorinic acid anthrone to norsolorinic acid may be catalyzed by *stcM*, a monooxygenase ortholog to *hypC* that converts norsolorinic acid anthrone to norsolorinic acid in *A. parasiticus* [43]. Norsolorinic acid is the first stable intermediate in the biosynthesis of sterigmatocystin and is converted into averantin by StcE, reducing the hexanoate ketone to an alcohol [41,55,56].

Yabe *et al.* [55] showed that 5′-hydroxyaverantin is a step towards aflatoxin in *A. parasiticus* and a later study showed that the oxidation of averantin into 5′-hydroxyaverantin is catalyzed by StcF [57]. The conversion of 5′-hydroxyaverantin to 5′-oxyaverantin is likely catalyzed by StcG [39,44]. In a study in *A. parasiticus* by Yabe and co-workers it was shown that both (1′*S*, 5′*S*)- and (1′*S*, 5′*R*)-hydroxyaveratin are formed in the conversion of averatin to 5′-oxyaveratin [58]. Disruption of *aflH* in *A. parasiticus* resulted in the accumulation of 5′-hydroxyaverantin, however, small amounts of *O*-methylsterigmatocystin present suggested that other enzymes may be involved in the reaction [44,59]. The gene(s) responsible for the conversion of 5′-oxyaverantin to averufin have not been identified [39,60].

Individual disruption of StcB and StcW resulted in elimination of sterigmatocystin and accumulation of averufin, indicating that both enzymes catalyze the conversion of averufin to 1-hydroxyversicolorone [57]. It was not possible for the authors to determine why two monooxygenases were required for this reaction step [57]. No gene products have been identified as being responsible for the conversion of 1-hydroxyversicolorone to versiconal hemiacetal acetate. StcI is thought to catalyze the reaction from 1-hydroxyversicolorone to versiconal based on studies of the ortholog AflJ in *A. parasiticus*, though other genes capable of this reaction may be present in *A. nidulans* [44,61].

Deletion of *stcN* did not result in the production of sterigmatocystin or other intermediates [44]. However, StcN show homology to AflK and the versicolorin B synthase, Vbs, in *A. parasiticus*, indicating that the biosynthetic step from versiconal to versicolorin B may be catalyzed by StcN [39,44,62]. StcL was shown by Kelkar *et al.* [63] to catalyze the conversion of versicolorin B to versicolorin A. Inactivation of *stcL* resulted in accumulation of dihydrosterigmatocystin, leading to a branching of the sterigmatocystin biosynthesis as seen in the aflatoxin biosynthesis. Addition of versicolorin A to the mutant gave production of sterigmatocystin, and that indicated that this enzyme functions before versicolorin A [63].

Keller and co-workers [64,65] showed that StcU and StcS are involved in the conversion of versicolorin A to demethylsterigmatocystin. Individual disruption of *stcU* and *stcS* led to the accumulation of versicolorin A and eliminated production of sterigmatocystin in *A. nidulans* [64,65]. Henry and Townsend [45] studied the same step in the aflatoxin biosynthesis in *A. parasiticus* and proposed an oxidation-reduction-oxidation mechanism, involving at least a ketoreductase AflM and a monooxygenase AflN, orthologs to *stcU* and *stcS*, respectively. Ehrlich *et al.* [46] and Cary *et al.* [47] identified two enzymes, AflY, a Baeyer-Villiger oxidase and AflX, an oxidoreductase, to be involved in the conversion of versicolorin A to demethylsterigmatocystin in *A. parasiticus* and *A. flavus*, respectively. *aflX* and *aflY* are homologous to *stcQ* and *stcR*, which suggests that these genes might be involved in the biosynthetic step from versicolorin A to demethylsterigmatocystin [46,47].

The final step in the biosynthesis of sterigmatocystin is the methylation of demethylsterigmatocystin catalyzed by StcP [66]. The conversion of sterigmatocystin to aflatoxin involves two additional biosynthetic steps; an *O*-methylation of sterigmatocystin by *aflP* followed by involvement of *aflQ* to produce aflatoxin [39]. Slot and Rokas have recently showed that the sterigmatocystin gene cluster in *Podospora anserina* was horizontally transferred from *Aspergillus*, which shows that transfer of large metabolite clusters between fungi are possible [67].

## 4. Aspyridone

**Figure 5:**
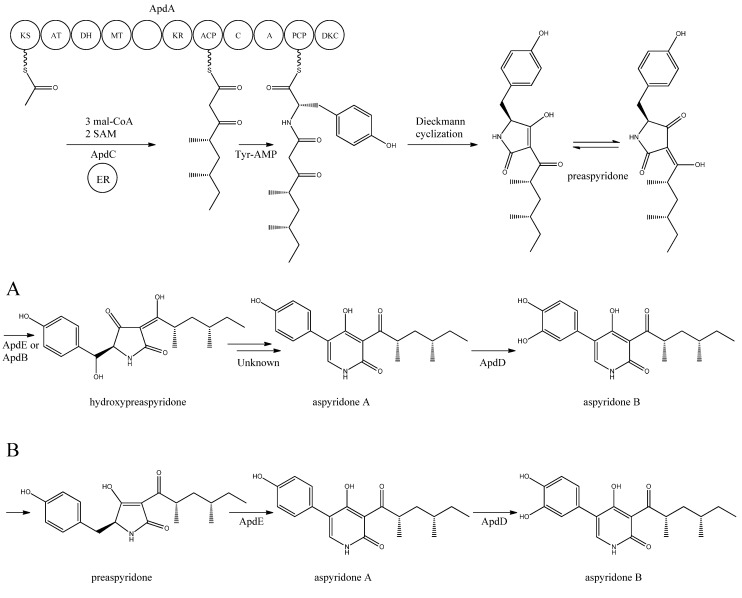
Proposed biosynthesis of aspyridone A and B. (**a**) Based on proposed biosynthesis by [68,72,73]; (**b**) Based on results by Halo *et al.* [78] Multiple arrows indicate that the number of enzymatic steps is unknown.

Aspyridone is a PK-NRP hybrid and a fascinating example on how PK-NRP compounds in *A. nidulans* can be assembled from the activity of a single fusion enzyme. The aspyridones have shown to display moderate cytotoxicity [68]. The responsible gene cluster was discovered by Bergmann and co-workers [68] using a genome mining approach. Using the *Aspergillus* genome sequence, they identified a SM gene cluster, which contained a putative TF (AN8414/*apdR*) that the authors hypothesized could trigger activation of the genes in the cluster. Accordingly, the authors overexpressed the TF under the control of an inducible *alcA* promoter by integrating it randomly in the genome. In agreement with the hypothesis, it was demonstrated by Northern blot analysis that six of the nearest neighbor genes were up-regulated in this strain on inductive medium, and that the aspyridones and two intermediates or shunt products could also be detected. Prediction of the catalytic potential for the six upregulated genes (*apdA, apdB*, *apdC*, *apdD*, *apdE,* and *apdG*) combined with the structure of the accumulating compounds allowed the authors to propose a model for the biosynthesis of aspyridones including an assignment of the involved enzymes (Figure 5).

*apdA* was deleted by Chiang *et al.* [69] and confirmed to be involved in aspyridone biosynthesis as reported by Bergmann *et al.* [68].

The structure of aspyridone A and B suggested that their synthesis involved both PKS and NRPS activity. Indeed, analysis of the AN8412 structure revealed domains characteristic for a HR-PKS as well as NRPS in one ORF spanning more than 11 kb. This is a special subclass of reducing PKSs, where the PKS has been directly fused with a single NRPS module at the C′-terminal end. This architecture allows for the incorporation of amino acids or carboxylic acids into the carboxylic end of the growing PK chain. Only one of these fusion enzymes has been found in *A. nidulans*, but has been reported in other fungi [70,71]. Since AN8412 is the first enzyme to act in the pathway, the gene was named *apdA*. ApdA catalyzes the assembly of the PK-amino acid backbone of the aspyridones by three Claisen condensations of malonyl-CoA, and KR-DH-ER-MT carries out full reduction of the β-keto and the methylations, which are required. However, as ApdA lacks a functional ER domain, the ER activity is most likely provided by ApdC, a homolog to an enoyl reductase (LovC) from the lovastatin biosynthetic gene cluster [68]. The resulting triketide is transferred to the NRPS module, where it is linked to tyrosine [68]. Bergmann and co-workers listed the domains through protein homology in the NRPS as condensation (C), adenylation (A), peptidyl carrier protein (PCP) and reductase domain (RED).The release of the PKS-NRPS hybrid product was proposed to be a NADPH-dependent reductive release followed by an intramolecular Knoevenagel condensation and enzymatic oxidation [68]. Biochemical studies of the role of ApdA and ApdC in the biosynthetic pathway of the aspyridones have been performed by Liu *et al.* [72] and Xu *et al.* [73]. Liu and co-workers [72] defined the NRPS module as C-A-T-R with the latter two being thiolation and reductase, which is an alternative to the more frequent C-A-T-TE found in these modules. However, this reductase domain (R*) in the NRPS module of ApdA is not the standard SDR superfamily dehydrogenase since tyrosine in the Ser-Tyr-Lys catalytic triad is mutated suggesting a redox-independent condensation reaction and the release of a tautomer of preaspyridone from ApdA by a Dieckmann cyclization, which was first shown by Halo and co-workers [74]. This result has been confirmed by Xu *et al.* who expressed the *apdA* and *apdC* genes in *Saccharomyces cerevisiae* and *Escherichia coli*, respectively [73]. The purified enzymes (ApdA and ApdC) were incubated in the presence of cofactors and building blocks and the predominant product was preaspyridone [73].

The formation of preaspyridone into aspyridone A and B was proposed by Bergmann *et al.* [68], (and outlined in Figure 5a) using the predicted functions of the remaining genes of the *apd* gene cluster. The proposed biosynthesis involved ApdB and ApdE which shows similarity to cytochrome P450 oxygenases and cytochrome P450 alkane hydroxylases, respectively, and were believed to catalyze the formation of hydroxypreaspyridone [68]. Based on the study of pyridone rearrangement in metabolites related to aspyridone it was suggested that ApdE or ApdB were involved in the pyridone rearrangement [68,75,76,77]. Moreover, aspyridone A was hypothesized to be converted into aspyridone B by ApdD, a putative FAD-dependent monooxygenase, which is related to other ring hydroxylases [68]. Aspyridone has a similar structure to other pyridines isolated from fungi, e.g., tenellin whose biosynthetic gene cluster also has been identified [75,76]. The proposed biosynthesis of aspyridone was, as described above, based on predicted gene functions and not isolated intermediates. However, a study on the biosynthesis of the related metabolite tenellin by Halo and co-workers [78] showed that the suggested biosynthesis may be incorrect and an alternative biosynthesis was suggested (as shown in Figure 5b). In this biosynthesis preaspyridone is not converted into hydroxyaspyridone but ring expanded by ApdE to aspyridone A similar to the biosynthesis of tenellin [78]. Halo *et al.* also showed that the hydroxylated metabolite of pretenellin is a shunt metabolite as it could not be converted into tenellin.

## 5. Emericellamides

The emericellamides are other examples of hybrid compounds that are formed between PKs and NRPs. In this case the biosynthesis requires a PKS and a NRPS rather than a fusion PKS-NRPS as used in the production of the aspyridones. Emericellamides are cyclic depsipeptides and a total of five variants, A, C–F, of these metabolites have been found in *A. nidulans*. Initially, emericellamide A and B were isolated and described from an unidentified marine-derived *Emericella* strain in a screen due to their antibacterial activity against methicillin-resistant *Staphylococcus aureus* [79].

In order to discover novel natural products, Chiang and co-workers [69] searched the genome sequence of *A. nidulans* for NRPS gene candidates. Subsequently, six of these genes were randomly chosen and deleted by gene targeting. One of the resulting mutants, AN2545Δ, showed a metabolite profile where emericellamide A was missing. Furthermore, HPLC profiles and dereplication using mass spectrometry and database searches revealed four additional compounds, which disappeared in the mutant metabolite profile. Since these compounds had not previously been described in *A. nidulans*, they were purified and their structures solved by NMR analysis revealing that they were novel analogues of emericellamide A and B, thus named emericellamide C–F [69].

To investigate whether AN2545, now called *easA*, defines a gene cluster encoding all necessary enzymatic activities in the emericellamide biosynthetic pathway, the genes from AN2542 to AN10325, a total of ten, were deleted [69]. Most of these gene deletions did not affect emericellamide production as judged by LC-MS analysis, demonstrating that they do not participate in the biosynthesis. However, the emericellamides were absent in four of the deletion strains, now named *easA-easD*, indicating that these genes are involved in the pathway.

**Figure 6:**
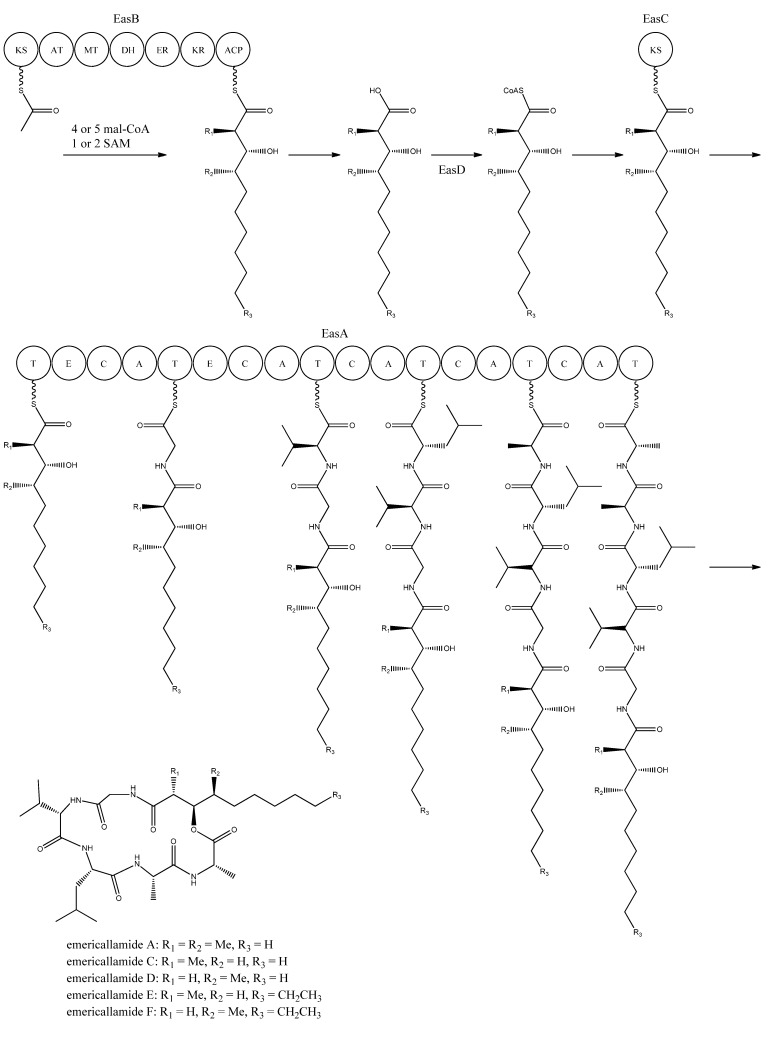
Proposed biosynthesis of the emericallamides. The order of the methyltransferase (MT) and dehydratase (DH) domain as suggested by Chiang *et al.* [69],however a BLASTp analysis suggests a swapping of the MT and DH domains. The NRPS, EasA, contains 18 (T, E (epimerization), C, A) domains grouped into five modules.

Bioinformatic analysis of the three additional genes suggested that they all encode activities that are relevant for emericellamide biosynthesis. Specifically, *easB* (AN2547), a PKS, *easC* (AN2548), an acyl transferase, and *easD* (AN2549), an acyl-CoA ligase. Based on these putative activities, the authors proposed a biosynthetic pathway for emericellamide production (Figure 6). In this model, the biosynthesis is initiated by EasB, a HR-PKS composed of the domains KS-AT-MT-DH-ER-KR-ACP. Since the PK component of the different emericellamide variants differ with respect to chain length and methylation pattern, it indicates that iterativity of this PKS is flexible [69].

Next, the PK carboxylic acid is converted to a CoA thioester by the acyl-CoA ligase, EasD, loaded onto the acyltransferase EasC, and then transferred to the thiolation (T) domain of EasA. This NRPS is a multi-modular enzymatic assembly containing 18 domains grouped into five modules. Among those, the authors propose that the first T domain is responsible for accepting the incoming PK from EasC (Figure 6). Moreover, the remaining domains fit well with the fact that five amino acids are incorporated into emericellamides. The authors note that this NRPS does not contain a TE domain at the end of module 5, indicating that this enzymatic activity is not necessary for cyclization of the emericellamides [69].

## 6. Asperthecin

Asperthecin is a PK compound that was first isolated from *A. quadrilineatus* by Howard and Raistrick in 1955 [80], however, the structure was not fully determined until six years later [81]. Initially, various chemical derivatizations and spectroscopic data determined a partial structure of asperthecin [80]. Neelakantan and co-workers [82] reduced the number of possible structures to two, and further derivatizations of asperthecin by Birkinshaw and Gourlay resulted in the final structure [81]. *A. quadrilineatus* is a member of the *A. nidulans* group, therefore Howard and Raistrick [80] extended the search of asperthecin to additional members of the *A. nidulans* group. No other was as rich in asperthecin production as *A. quadrilineatus*, yet small amounts of crystallized asperthecin could be obtained from cultures of *A. nidulans* and *A. rugulosus* indicating that production of asperthecin was possible in other aspergilli [80].

About 50 years later, Szewczyk and co-workers [83] used a molecular genetics approach to find the gene cluster responsible for the production of asperthecin in *A.nidulans*. Since many aspects of the regulation in the polyketome were unknown, the authors speculated whether sumoylation had an effect. SUMO is a small ubiquitin-like protein which is post-translationally added to proteins in the cell, as it plays a role in regulating transcription. *A. nidulans* contains one SUMO encoding gene, *sumO* [84], deletion of which led to a decrease in the production of SMs such as austinol, dehydroaustinol, and sterigmatocystin, and an increase in the production of a metabolite identified to be asperthecin, whereas the production of emericellamides were not affected [83]. Due to the aromatic structure of asperthecin, Szewczyk *et al.* [83] studied the domain prediction in 27 putative PKS-protein sequences using the *A. nidulans* genome sequence and available tools, in order to identify potential producers of non-reduced PKs. Ten NR-PKSs were identified and a deletion series of all NR-PKS genes was performed in the *sumO*∆ background [83]. While nine of the PKS-deletion strains still produced asperthecin, the AN6000 (*aptA*) PKS-deletion strain failed to synthesize asperthecin [83]. With the notion that most end compounds in PK biosynthesis are made by a clustered gene collective, six candidate genes surrounding *aptA* were picked in an attempt to identify the *apt* biosynthetic cluster. Two of these genes, *aptB* (AN6001) and *aptC* (AN6002), were found to be required for asperthecin production [83]. One strain (AN5999Δ) had a significantly lower production of asperthecin compared to the reference strain, but asperthecin was still present in the metabolite extracts, and as the strain showed poor growth, it was not included in the *apt* gene cluster.

**Figure 7:**
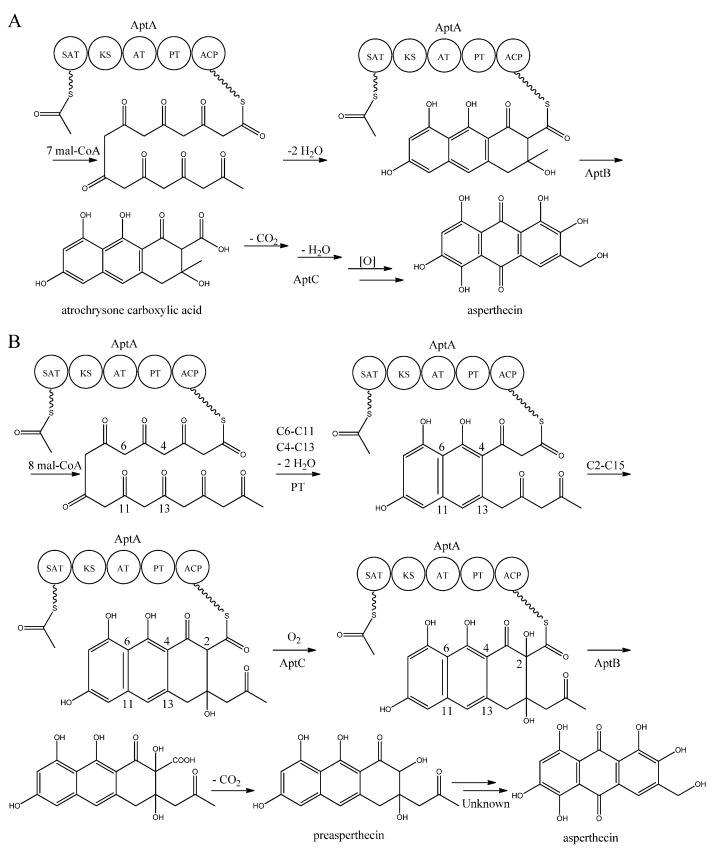
Proposed biosynthesis of asperthecin (**a**) As suggested by Chiang *et al.* [86]; (**b**) Based on proposed biosynthesis by Li *et al.* [87]. AptA contains SAT, KS, AT, PT and ACP domains. Multiple arrows indicate that the number of enzymatic steps is unknown.

Interestingly, AptA was shown to have SAT-KS-AT-PT-ACP domains, but lack a TE/CLC domain [83,85,86,87,88]. Independent groups have used this case as a model system to study the mode of PK release, and two alternating mechanisms for the biosynthesis of asperthecin are shown in Figure 7 [86,87]. The first model suggests the formation of the PK backbone by condensations of one acetyl-CoA and seven malonyl-CoA units [86], with the β-lactamase AptB releasing the octaketide from AptA [83]. This assumption was based on a study by Awakawa and co-workers [85] in *A. terreus*, where there was a release of atrochrysone carboxylic acid from the atrochrysone carboxylic acid synthase (ACAS) lacking a TE/CLC domain, in the presence of the atrochrysone carboxylic ACP thioesterase (ACTE), a member of the β-lactamase superfamily [85]. The unstable atrochrysone carboxylic acid then undergoes a series of reactions; decarboxylation, dehydration, and various oxidations where the monooxygenase AptC is believed to be involved, and in the end yielding asperthecin [86].

In another approach, Li *et al.* [87] introduced *aptA*, *aptB*, and *aptC* into *S. cerevisiae*, which resulted in the production of a nonaketide (here called preasperthecin), and not the octaketide as proposed by Chiang *et al.* [86]*.* Expressing *aptA* and *aptB* without *aptC* resulted in a product identical to preasperthecin except for the lack of C2-OH, confirming that AptC is responsible for this step [87]. Expression of *aptA* and *aptC* alone did not lead to the production of preasperthecin or any other traceable compounds, confirming that AptB is needed for release of the PK from AptA [87]. These results were confirmed by an *in vitro* assay after expressing *aptA*, *aptB* and *aptC* in *E. coli* [87]. Further insight into AptA functionality came from expressing the AptA-PT domain in *E. coli*, and combining it with the *Gibberellafujikuroi* PKS4, which can produce nonaketide products *in vitro* [88]. The experiment revealed that AptA-PT can catalyze C6-C11 cyclization, and most likely also the C4-C13 cyclization. Further, a spontaneous C2-C15 cyclization was followed by a C1-C17 esterification [88]. The *apt* gene cluster appears likely to consist of additional genes, which are responsible for the conversion of preasperthecin into asperthecin.

## 7. Asperfuranone

Asperfuranone is an example of a novel PK metabolite discovered through genetic mining in *A. nidulans*, as this compound had not previously been reported in the literature before Chiang and co-workers [89] in 2009. Asperfuranone was later shown to possess bioactive properties as it inhibited proliferation of human non-small A549 cancer cells [90]. Investigating the loci containing putative PKS gene clusters, Chiang and co-workers [89] noticed that a NR-PKS gene (AN1034, *afoE*) and a HR-PKS gene (AN1036, *afoG*) were located close to each other on chromosome VIII. Since no products had ever been detected from activity of this locus, and due to the rare constellation of two neighbor PKSs, the authors speculated whether a novel metabolite could be revealed. A putative transcriptional activator (AN1029, *afoA*) was found near the PKS and the authors replaced the upstream sequence of *afoA*, estimated to be the native promoter, with the inducible *alcA* promoter [89]. This indeed turned on the expression of the cluster, since asperfuranone and a precursor metabolite were detected. The structure of asperfuranone was determined based on one- and two-dimensional NMR experiments and the absolute configuration by a modified Mosher’s method, whereas the precursor preasperfuranone had already been determined in the literature [89,91]. With these two compounds being identified, a gene-deletion strategy was performed to map the other genes assigned to the *afo* gene cluster, which involved twelve surrounding genes including the two PKSs [89]. Four deletion strains *afoD∆*, *afoE∆*, *afoF∆*, and *afoG∆* fully eliminated asperfuranone production whereas *afoB∆* and *afoC∆* strongly reduced production of asperfuranone [82]. The deletions confirmed that both *afoE* and *afoG* were responsible for the production of asperfuranone, and that the deletion of *afoD*, encoding a putative hydroxylase, resulted in the production of preasperfuranone [89]. Deletion of *afoB* reduced the production of asperfuranone and due to a high homology to efflux pumps, Chiang and co-workers [89] suggested that it was responsible for the transport of asperfuranone out of the cell.

**Figure 8:**
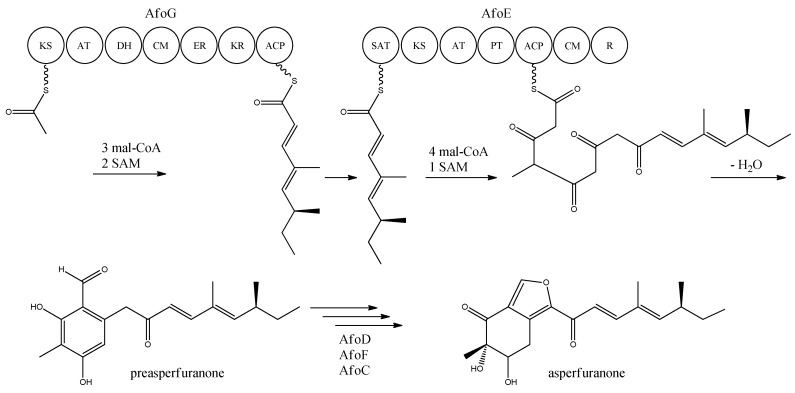
Proposed biosynthesis of asperfuranone. The highly reducing (HR)-PKS, AfoG, contains KS, AT, DH, CM, ER, KR, and ACP domains whereas the NR-PKS, AfoE, contains SAT, KS, AT,PT, ACP, CM , and R domains. The only intermediate isolated in the biosynthesis is preasperfuranone [89]. Multiple arrows indicate that the number of enzymatic steps and reaction order is unknown.

With the gene cluster and predicted functionalities of the gene products defined, a biosynthetic pathway of asperfuranone was proposed (Figure 8) [89]. The assembly of the primary reduced tetraketide is synthesized by AfoG from one acetyl-CoA, three malonyl-CoA, and two SAM. The tetraketide is transferred to the SAT domain of AfoE and extended with four malonyl-CoA and one SAM [89]. The octaketide is released from AfoE after aldol condensation and reductive release from a C-terminal reductase (R) domain, which resembles a reductive release mechanism to generate the aldehydes described by Bailey *et al.* [92], forming the aldehyde preasperfuranone [89]. The biosynthetic steps from preasperfuranone to asperfuranone are uncharacterized and the suggestions are not based on identified metabolites [89]. Accumulation of preasperfuranone in the *afoD*Δ suggested AfoD to be the next enzyme in the biosynthesis of asperfuranone. The deletions of *afoF*, encoding a putative FAD/FMN-dependent oxygenase and *afoC*, initially believed to code for a homologue to citrinin biosynthesis oxidoreductase, did not reveal any intermediates, the order of reactions and the exact enzymatic functions for AfoF and AfoC have not been determined. In *afoC∆*, the production of asperfuranone was not fully eliminated, which Chiang and co-workers [89] suggested could be due to other enzymes catalyzing the reaction, however less efficiently.

Other puzzling discoveries have been made in connection to the asperfuranone production. When trying to activate a cryptic NRPS gene cluster containing two NRPSs, *inpA* (AN3495) and *inpB* (AN3496), by overexpression of a regulatory gene, *scpR* (AN3492), Bergmann *et al.* [93] also activated asperfuranone. This is an interesting example of a regulatory gene located on chromosome II that activates the *afo* cluster located on chromosome VIII [93]. Lui *et al.* [94] have attempted to engineer the production of a new metabolite by swapping the SAT domain of AfoE with the StcA-SAT. This led to the production of a new metabolite though having the same length as the native AfoE product, asperfuranone [94].

## 8. Monodictyphenone/Emodin

The PK monodictyphenone was first reported in *A. nidulans* in 2005 [95] and the genes behind the production of monodictyphenone were mapped four years later [96]. This discovery not only enabled the establishment of a biosynthesis model for monodictyphenone in *A. nidulans*, it has subsequently revealed that more than ten different stable products among different classes of related polyketides can be linked to monodictyphenone biosynthesis [11,96,97,98]. These metabolites count monodictyphenone, emodin and the emodin derivates 2-hydroxyemodin, 2-aminoemodin, ω-hydroxy emodin, and emodic acid. Moreover, the arugosins and prenyl-xanthones are also coupled to the pathway [11,98]. The compound emodin has been studied for more than a century [99], and is an anthraquinone found in a wide array of both plants and fungi [100,101]. Emodin and several derivatives (e.g. emodic acid) have been shown to possess anti-bacterial and cancer preventive properties [102,103,104,105,106].

The presence of the SM clusters in silent areas of chromosomes, e.g. near telomeres and centromeres, suggests that chromatin remodeling factors can influence the expression of genes responsible for SMs. As rationalized by Bok and co-workers [96], removal of histone-tail methylation could open heterochromatic regions for transcription. The authors deleted an ortholog, *cclA*, to the yeast *BRE2* gene, encoding an enzyme partner of the COMPASS transcriptional regulator complex conserved in eukaryotes, which rendered *A. nidulans* defective in di- and trimethylation of lysine 4 of the histone 3 tails (H3K4). The *cclA* deletion was established in a mutant strain, *stcJ*Δ, to avoid interference of high amounts of sterigmatocystin in purification of other metabolites. The effect was striking as the loss of CclA in HPLC analysis showed an altered chemical landscape compared to the *stcJ*Δ reference [96].

As the compounds appearing were UV-active suggesting high conjugation likely due to aromaticity, the ten NR-PKSs investigated in the asperthecin and asperfuranone studies were individually deleted in the *cclA*Δ *stcJ*Δ double deletion background. This screen revealed six products that all were linked to one PKS, AN0150 (*mdpG*) [96]. Delineation of the cluster was achieved by inspecting the genome sequence for possible cluster candidates followed by Northern blotting for gene-expression analysis in the *cclA*Δ *stcJ*Δ, where the products were detected. The cluster was found to span 12 putative ORFs (AN10021-AN10023 (*mdpA-L*)) [97] of which two genes AN0147 (*mdpD*) and AN10035 (*mdpI*), did not show altered expression from the reference [96,97]. The *mdp*-cluster candidates were also deleted in the *cclA*Δ *stcJ*Δ mutant strain to confirm the expression analysis data and to draw the borders of the cluster [97]. The authors suggest that two transcriptional activators are present within the cluster; MdpE as a main activator (homologue to AflR) and that MdpA is a co-activator. The *mdp* locus is located near the telomere of chromosome VIII, and activation of the genes in the *cclA*Δ strain supports the hypothesis of epigenetic regulation in these areas through chromatin remodeling [96].

Two groups cultivated *A. nidulans* on complex growth media, which revealed six additional metabolites. First Sanchez *et al.* [98] discovered that emericillin, variecoxanthone A, shamixanthone, and epi-shamixanthone were also products of the *mdp* cluster, and subsequently Nielsen and co-workers [11] added arugosin A and H to the pathway. Since these PKs are prenylated, a BLAST search of the *A. nidulans* genome sequence was performed and pathway-candidate genes were deleted. Two prenyl transferases encoded by *xptA* and *xptB*, and one neighbor GMC oxidoreductase encoded by *xptC*, were found to be involved in the pathway, though they were located on other chromosomes than the *mdp* cluster (for cluster overview see Sanchez *et al.* [98]). This is an intriguing example of SM-cluster members located on more than one chromosome, however, prenyl transferases are known to have broad substrate specificity, and it is currently not known whether they are involved in other processes than prenyl-xanthone formation [98].

**Figure 9:**
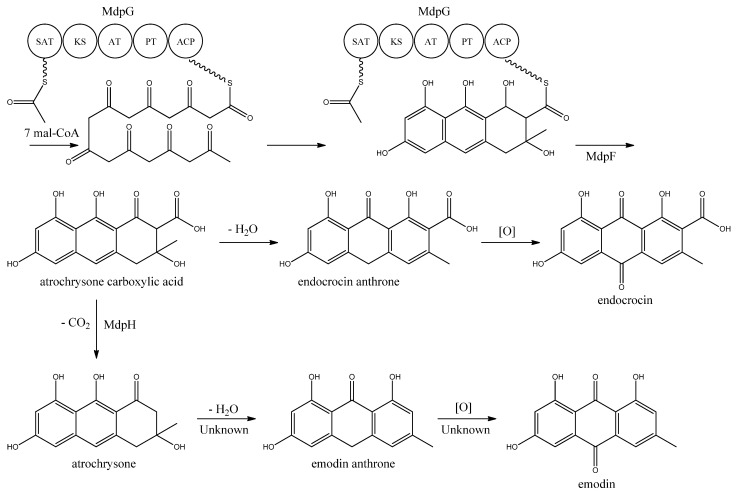
Proposed biosynthesis of emodin. MdpG contains SAT, KS, AT, PT, and ACP domains.

MdpG synthesizes the main PK backbone. Since MdpG lacks a CLC/TE domain, MdpF, a putative zinc dependent hydrolase, is believed to catalyze the release of the PK from MdpG [97]. The mechanism is believed to follow the case of ACAS and ACTE as introduced previously in the asperthecin section. Awakawa and co-workers [85] demonstrated that the direct product of the ACAS/ACTE is not emodin anthrone as proposed earlier [107,108], but more likely atrochrysone carboxylic acid (Figure 9). Atrochrysone carboxylic acid was not observed *in vitro*, instead the decarboxylated product atrochrysone was the major product in the assays and therefore proposed to be an intermediate to emodin, as suggested by Couch and Gautier [109]. Conversion of atrochrysone to emodin requires dehydration (forming emodin anthrone) and a final oxidation (Figure 9), however, Awakawa and co-workers [85] observed small amounts of both emodin anthrone and emodin *in vitro* showing that these reactions may occur non-enzymatically. Based on these observations, Chiang and co-workers [97] proposed that *mdpH* encodes a decarboxylase, catalyzing the conversion of atrochrysone carboxylic acid to atrochrysone. The deletion of *mpdH* resulted in accumulation of a shunt product endocrocin produced via endocrocin anthrone. Enzymes responsible for dehydration of atrochrysone or modification of emodin into the observed derivatives have not yet been identified.

The first stable intermediate following emodin towards the prenyl-xanthones is monodictyphenone [97,98], and gene-deletion studies points to at least the five following enzymes are involved; a dehydratase (MdpB), a ketoreductase (MdpC), a glutathione S transferase (MdpJ), an oxidoreductase (MdpK), and a Baeyer-Villiger oxidase (MdpL) [97]. The mechanism has been proposed to be analogous to the conversion of versicolorin A to demethylsterigmatocystin which is known to proceed through oxidation-reduction-oxidation catalyzed by a cytochrome P450 monooxygenase (VerA) and a ketoreductase (StcU) [45,65,110]. However, none of the above mentioned Mdp enzymes appear to be homologous to VerA, and the role of the individual enzymes has not been investigated further [97].

**Figure 10:**
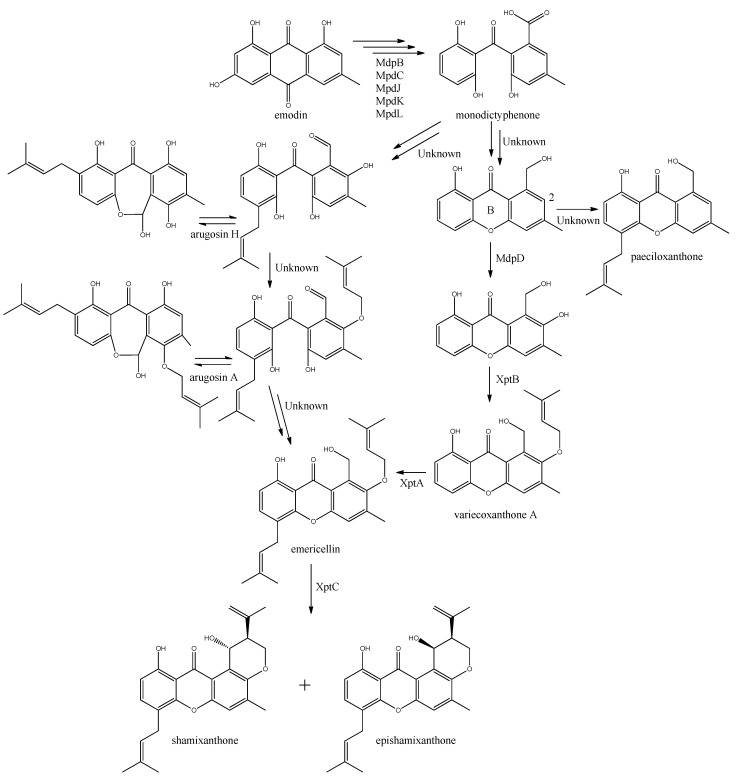
Suggested biosynthesis of the shamixanthons from emodin. Multiple arrows indicate that the number of enzymatic steps are unknown.

The biosynthesis of the six monodictophenone derived metabolites is based on hydroxylation (MdpD), C-prenylation (XptA), *O*-prenylation (XptB), and carboxylic acid reduction (unidentified enzyme) [11,98]. Central in the pathway is the hydroxylation of C2 in monodictyphenone accompanied by reduction of the carboxylic acid. The carboxylic acid is suggested by Sanchez and co-workers [98] to be reduced to a hydroxy group, the B-ring is closed by dehydration and the intermediate is O-prenylated at C2 to yield variecoxanthone A, which in turn is C-prenylated to emericillin (Figure 10). The final known step in prenyl-xanthone biosynthesis gives rise to the stereoisomers shamixanthone and epishamixanthone and is catalyzed by XptC [98]. Alternatively, Nielsen and co-workers include synthesis of arugosins by partially reducing the carboxylic acid to an aldehyde, followed by C-prenylation, yielding arugosin H and O-prenylation to give arugosin A. Subsequent reduction of the aldehyde to a hydroxyl group, and ring closure by dehydration then gives emericillin and shamixanthones [11].

## 9. Orsellinic Acid

In addition to the *mdp* cluster, the loss of CclA also led to the discovery of another gene cluster driven by an NR-PKS [96]. Two PK products, the cathepcin K inhibitors F-9775A and F-9775B, first isolated from *Paecilomyces carneus* [111], were detected and mapped to AN7909. Following this discovery, Schroeckh and co-workers [112] found the primary metabolite from AN7909 (*orsA*) to be orsellinic acid, an archetypal metabolite [113]. Moreover, the metabolite lecanoric acid typically found in lichens and produced by mycobiots such as *Umbilicaria antarctica* [114] was linked to OrsA [112]. Following the initial observations, the number of detected metabolites from the *orsA* gene cluster has expanded to gerfelin, a C10-deoxy-gerfelin, diorcinol, orcinol, cordyol C, and violaceol I and II [115,116]. The biosynthetic activities of the *orsA* cluster are as yet not elucitated, and this illustrates the need for applying different eloquent strategies to trigger production of these metabolites.

The deficiency in methylation of H3K4 in the *cclA*Δ strain resulted in activation of both *mdp* and *ors* gene clusters. Expression analysis revealed that the annotated ORFs from AN7909-AN7915 were possible cluster members, hereby indicating candidates for a gene cluster [96]. The *ors* gene cluster, *orsB-orsE*, was identified by Schroeckh and co-workers [112] as four additional ORFs spanning AN7911-AN7914, which was confirmed by Sanchez *et al.* [115], who deleted all genes from AN7901 to AN7915. Interestingly, the neighbor PKS to the *ors* locus, AN7903, was deleted by Nielsen *et al.* [11] and the resulting strain failed to produce F-9775A and B like AN7909Δ under the conditions tested. Schroeckh and co-workers [112] defined *orsA-E* using gene-expression analysis through both an *Aspergillus* secondary metabolism array (ASMA) and relative expression analysis in quantitative reverse-transcriptase PCR (qRT-PCR). The induction of *orsA* was achieved by co-cultivating with a soil bacterium, *Streptomyces rapamycinicus* (initially named *S. hygroscopicus*) and extracting mRNA from the fungus [112]. This response on SM level was further investigated by Nützmann and co-workers [117]. Since the loss of H3K4 methylation induced gene expression in *ors* locus [96], the rationale was that the transcriptional activation of silent secondary-metabolism genes by acetylation of lysines on histone tails, especially H3K9, is equally important and the search for histone acetyl transferases (HATs) in the genome sequence was commenced [117]. Forty HATs were found and deleted, and only four proved to be essential. Of the 36 deletions of nonessential HATs in *A. nidulans*, the deletions of *gcnE* and *adaA*, both essential core parts of the multi-subunit Saga/Ada complex, an important complex for HAT activity in *A. nidulans*, significantly lowered the *ors* transcripts investigated [117]. Thus, Saga/Ada plays a role in the response to *S. rapamycinicus* and loss of this complex downregulated orsellinic acid metabolites, as well as sterigmatocystin, terrequinone, and penicillin [117].

Four additional orsellinic acid derived compounds were found in a defect COP9 signalosome (CSN) mutant strain [116]. The multiunit CSN complex is found in higher eukaryotes, albeit with different functional roles depending on the tissues. In *A. nidulans* the CSN is required for fruiting body formation and is not essential for asexual growth. By deleting *csnE*; orcinol, cordyol C, and violaceol I+II were produced, and the genes *orsA-orsE* were shown to be differentially expressed [116]. The link of the violaceol metabolites to *ors* was confirmed by Nielsen and co-workers [11] who applied an OSMAC strategy on their reference strain and compared this to their deletion library.

**Figure 11:**
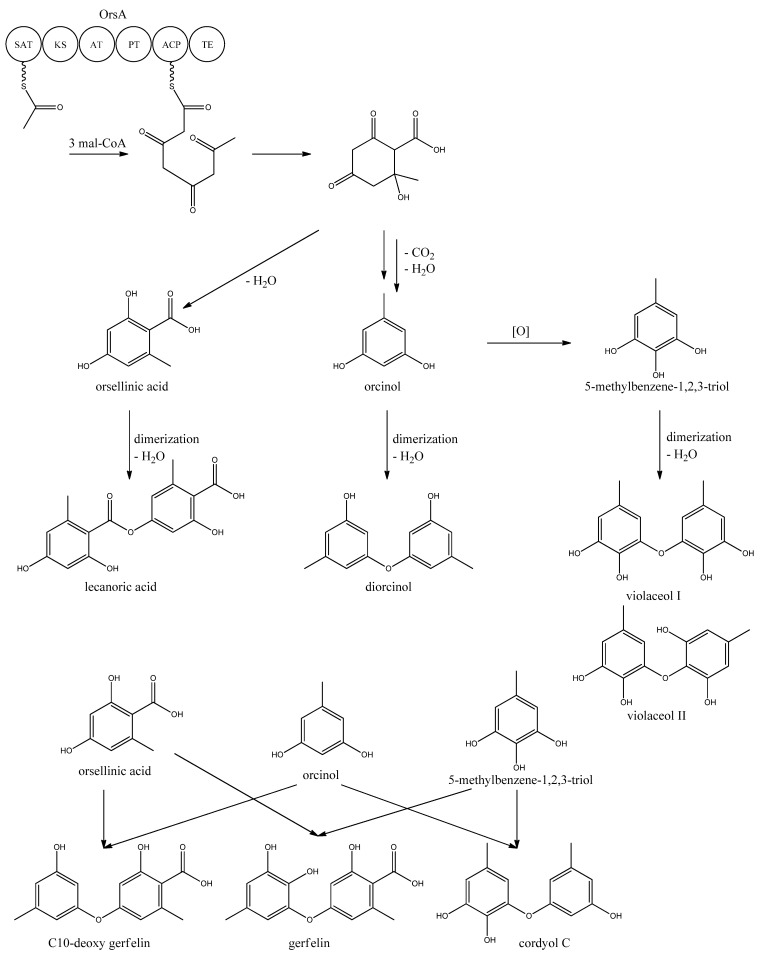
Proposed biosynthesis of orsellinic acid and its derivatives of orsellinic acid. The enzymes that catalyze the individual reactions in the biosynthesis of the metabolites are so far unknown and biosynthesis is proposed based on the observed metabolites.

**Figure 12:**
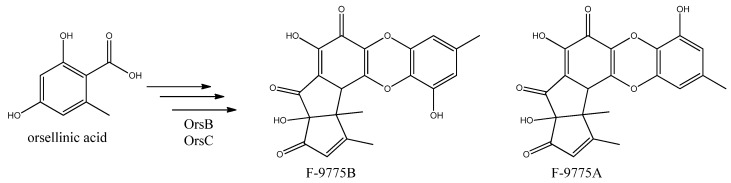
The proposed enzymes involved in the biosynthesis of F-9775A and F-9775B of the figure here.

Very little is known about the biosynthesis of the metabolites of the *ors* locus. One acetyl-CoA and three malonyl-CoA units can yield a C8 aldol intermediate, and as proposed by Nielsen *et al.* [11], this can lead to the tetraketide orsellinic acid through loss of water and enolization and to the C7 compound orcinol by decarboxylation and enolization. Oxidation of orcinol in the para position then leads to 5-methyl-benzene-1,2,3-triol which is believed to either dimerize with the loss of water to give violaceol I and II, Figure 11, or to give F-9775A+B, Figure 12, in an unknown series of synthesis steps. Another outcome is the formation of lecanoric acid by dimerization of orsellinic acid. Though the steps in the pathway have been hypothesized, most steps are not accounted for. It has been reported that OrsA having the domains SAT-KS-AT-PT-ACP-TE is responsible solely to form orsellinic acid. OrsA, OrsB, and OrsC seem to be sole responsible for F-9775A+B formation. Moreover, it has been shown that gerfelin and a C10-deoxy derivative of gerfelin accumulate in *orsB*Δ, whereas diorcinol was found in high amounts in the *orsC*Δ strain [115]. Gerfelin, C10-deoxy gerfelin and cordyol C are all dimers built up of two of the three suggested monomer units, orsellinic acid, orcinol and 5-methylbenzene-1,2,3-triol.

Recently Scherlach and co-workers [118] continuously cultivated *A. nidulans* under nitrogen-limitation and carbon-limitation. At nitrogen limiting conditions in continuous cultivations two novel products, denoted as spiroanthrones, were found. They could not be detected at batch cultivation. The metabolites were based on anthraquinone and orsellinic acid derived phenols. The induced expression of both *mdpG* and *orsA* confirmed increased activity under the N-limiting continuous cultivation conditions.

## 10. Austinol and Dehydroaustinol

The meroterpenoids austinol and the related compound dehydroaustin were first isolated from *A. ustus* by Simpson and co-workers in 1982 [119], where the structure of austinol was elucidated by ^1^H and ^13^C NMR. Austinol and dehydroaustinol are just two examples out of many meroterpenoids that are derived from 3,5-dimethyl orsellinic acid as presented in the excellent review by Geris and Simpson [120].

The two austinols were detected for the first time in *A. nidulans* four years ago [121], where it was further substantiated that austinol was indeed of partly PK origin. Deletion of the phosphopantheteinyl transferase (PPTase) *cfwA*/*npgA* in *A. nidulans* resulted in a strain that among many other compounds did not produce austinol and dehydroaustinol [121]. The PPTase is responsible for attaching the phosphopantetheine moiety to the acyl carrier domain of the PKSs and NRPSs, thus it is an activator of the enzyme complexes. Hence, the abolition of austinol and dehydroaustinol production in the PPTase deficient strain strongly suggests a PK origin of these compounds.

In 2011, Nielsen and co-workers [11] discovered the PKS responsible for synthesis of the PK part of austinol and dehydroaustinol in *A. nidulans*. A deletion library of all 32 putative PKS genes in *A. nidulans* was created and screened using an OSMAC approach [122] to enable activation of different clusters on different media. One single strain deleted in AN8383 (*ausA*) failed to produce austinol and dehydroaustinol [11]. This discovery was supported by the introduction of a point mutation at the phosphopantetheine attachment site, thus abolishing activation of the enzyme by the PPTase to ensure the loss of austinols was not an indirect effect e.g., at chromatin level. The *ausA*Δ strain was complemented by re-introducing the *ausA* gene into the deletion strain. Introducing the gene under control of the inducible *alcA* promoter revealed that 3,5-dimethyl orsellinic acid (3,5-MOA) was indeed the precursor for austinol and dehydroaustinol in *A. nidulans*, as shown experimentally via labeling studies by Simpson and co-workers 20 years earlier [11,123,124].

**Figure 13:**
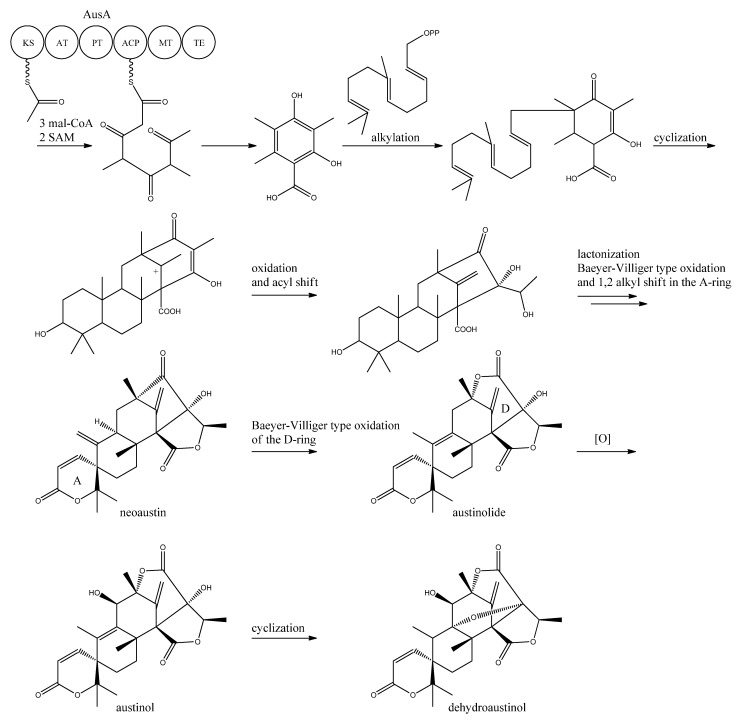
Proposed biosynthesis of austinol and dehydroaustinol. All genes in the biosynthesis of austinol and dehydroaustinol, except AusA, are unknown.

A more detailed understanding of the biosynthesis of the austinols has not been established yet. However, it is well established that 3,5-dimethyl orsellinic acid is synthesized from condensation of an acetyl-CoA unit with 3 malonyl-CoA units to form the PK backbone, which is methylated twice, catalyzed by AusA. The PK part is then alkylated with farnesyl pyrophosphate to form a transient intermediate (Figure 13), which can then act as a precursor for several similar meroterpenoids, such as andibenins, austin, berkeleyones and andrastins [120]. Recently we tentatively identified neoaustin and austinolide in *A. nidulans* extracts by LC-MS analysis (unpublished data), which makes us hypothesize that the biosynthesis towards the austinols involves (i) oxidation and acyl shift in the D ring (ii) lactonization from the substituent groups of the D ring, a Baeyer-Villiger type oxidation and 1,2 alkyl shift in the A ring to give neoaustin. Neoaustin is subsequently oxidized in the D ring by another Baeyer-Villiger type oxidation to give austinolide that upon further oxidation and ring condensation leads to austinol and dehydroaustinol, Figure 13.

## 11. Concluding Remarks

Secondary metabolism represents chemical diversity and span in biological functionality to the extreme. As shown above, individual compound classes can even form hybrid molecules to other compound classes. There is a high commercial interest in discovery and utilization of SMs in general as drugs or additives, or to avoid mycotoxins in food and feeds. Mapping PK biosynthesis to genes in *A. nidulans* as presented in this review involves great complexity. One challenge is to find and activate the genes required to produce the compounds. As shown, it takes in-depth understanding of fungal biology, nutrient sensing, chromatin remodeling, as well as analysis on all levels from DNA to metabolites to unveil cryptic gene clusters and their products. Moreover, the majority of the pathways described in this review have been elucidated in a relatively short time span. This has been possible due to bioinformatics. The availability of the genome sequence, as well as resources and tools in, e.g., *Aspergillus* Comparative Database (ACD), *Aspergillus* Genome Database (AspGD), and the Central *Aspergillus* REsource (CADRE) have been key aids to perform the extensive genome mining.

The ability to predict enzymatic function based on gene sequences has proven fruitful in characterization of secondary metabolism, since this has revealed the location of e.g., PKS genes. Additionally, the presence of gene cluster specific TFs was utilized in activation of silent SM clusters both in the case of aspyridones and asperfuranone. The asperfuranone biosynthesis is moreover an example of a previously unknown compound, with a potential to be a novel drug, has been found in a well-known filamentous fungus. Another case of a metabolite with attractive properties is emodin, which has been known for more than a century, but just recently had the biosynthetic machinery uncovered in *A. nidulans*. Both asperthecin and the emericellamides were firstly discovered and isolated from less well described aspergilli, however, after the compounds were observed in *A. nidulans*, the candidates for responsible genes in biosynthesis were found in a few months. The study of conidial pigment biosynthesis in *A. nidulans* has contributed to our basic understanding of fungal development and PKS organization, and provided researchers with an easy assayable marker system for genetic studies. Interestingly, the structure of the final pigment(s) still remains unknown after more than 70 years of research, underlining the difficulties in elucidating structures of highly polymerized PKs.

Epigenetic regulation through chromatin remodeling has shown to be involved in activation of several SM gene clusters. Conserved chromatin remodeling factors have influenced both local activation of some SM clusters and more global responses within the genome. Gene clusters producing sterigmatocystin, orsellinic acid, emodins, and austinols have shown to respond to specific factors. The presence of SM producing genes outside gene clusters, e.g., in prenyl-xanthone production, is probably more common than observed so far. Moreover, cross-talk between pathways is frequently observed, as more pathways become known. This can open a discussion whether common pools of intermediates or enzymes can exist. In addition, the compartmentalization of SM production is an area to be explored. Furthermore, controlling compartmentalization of production as well as secretion will influence yields and downstream applications which are important factors for exploiting SM production commercially. Existing compounds can be modified genetically to add/remove chemical groups on existing drugs, mix moieties from different SM classes or species by for example domain swapping, and to considerably increase/abolish a specific production.

Altogether the recent uncovering of secondary metabolism in *A. nidulans* is an illustrative example of strong interdisciplinary efforts requiring strong expertise within chemistry, biology, microbiology, molecular genetics, protein chemistry, computer science, and engineering. Ultimately, the efforts described in this review can form the basis for uncovering of the specific biological roles of the chemical arsenal in the fungus.
